# The Burdens of Orthopedic Patients and the Value of the HEPAS Approach (Healthy Eating, Physical Activity, and Sleep Hygiene)

**DOI:** 10.3389/fmed.2021.650947

**Published:** 2021-05-04

**Authors:** Matteo Briguglio

**Affiliations:** IRCCS Orthopedic Institute Galeazzi, Scientific Direction, Milan, Italy

**Keywords:** aging, osteoporosis, post traumatic, sarcopenia, orthopedics, diet, healthy, physical activity, sleep habits, quality of health care

## “No Country for Old Men” (Joel and Ethan Coen, 2007)

Aging is accompanied by an inexorable decline in physiological reserves, with life-course determinants entangling with conditions that put a strain on both the body and mind. In the current research panorama, the concept of health restoration is often associated with the fast regaining of bodily abilities intended as physical and mental dynamisms (1, 2), echoing the movement Futurism of the early 1990's that despised the stasis of reality and exalted the beauty of speed (3). What slowly deteriorates has to be quickly corrected and there would be no room for old age nevertheless, slowing the body, the mind, and the society. Indeed, orthopedic care pathways are often referred to as accelerated pathways or “fast-tracks,” symbolizing the firm surgery times, the shortened hospital stay, and the early rehabilitation (4). However, it is a fact that the world is populated by old people. On the one hand, there is high life expectancy while on the other hand there exists a technological society in which most of the elderly have no place. Their physical frailty, mainly associated with poor nutrition and immobility, is inevitably aggravated by psychological and social connotations (5). The HEPAS (Healthy Eating, Physical Activity, and Sleep hygiene) is a multidisciplinary approach to support the physical and mental health of individuals at risk of/with neuropsychiatric diseases that was first presented by Italian and American experts from IRCCS Orthopedic Institute Galeazzi and Stanford University (6). The cornerstones of this program include food and nutrition education, the promotion of an active lifestyle, and indications to aid restful sleep. In its conceptual model, HEPAS may not only be translated from Neuropsychiatry to Orthopedics, but it could also be feasible in the various stages of orthopedic disease progression, being implemented during the phase of health promotion and prevention of musculoskeletal disorders, the phase of reduction of disease burden, and the phase of adverse outcome prevention. The aim of this opinion article is to discuss how HEPAS can be exploited in the journey of orthopedic patients, showing its potential value in perfectly matching the burdens that grip the old person.

## “Gravity” (Alfonso Cuarón, 2013)

Terrestrial gravity has designed the evolution of our musculoskeletal system under a constant force of 1 g, pointing to a finite bone mineral density. This is evident in the variance between those with heavy vs. light bodyweight (7) or between those who exercise vs. those who do not (8). Regardless of the gravitational force, nutrition plays a major role in sustaining musculoskeletal health and this has been clearly highlighted by studies on long stays in space (9). Regrettably, aging is commonly associated with weight loss and therefore reduced gravitational pressure on bones, malabsorption that couples with low nutrient intake, increased anabolic threshold that alters requirements, and monotonous dietary habits that reduce nutrient coverages. The resulting consequence is an aging individual dealing with a para-physiological musculoskeletal involution who embraces a bed-kitchen-sofa lifestyle and repeated naps/sleepless nights that expose to behavioral alterations and reduce the ability to cope with listlessness in meal preparation or everyday exercise (10). Targeted interventions favoring healthier dietary habits and a more active lifestyle have often been studied -albeit separately- in elderly individual at risk of/with osteosarcopenia or undergoing elective orthopedic surgery (11–13). However, the systematic integration of a comprehensive lifestyle approach like HEPAS has not yet been reported, and the prototypical community-dwelling old men is still being an individual of about 70 years of age suffering from nutritional deficiencies, sarcopenia, osteoporosis, and cardiovascular diseases (14, 15). Importantly, various syndromic or pathological combinations can give rise to more complex prototypes, such as the obese sarcopenic or the multi-frail (16). For these individuals, gravitational acceleration is a double-edged sword because, while keeping the bones dense, it pushes down the body of those who are unstable, fracturing the fragile bones.

## “Fracture” (Gregory Hoblit, 2007)

Once in the hospital, the old patient with a fracture is exposed to many hazards if not carefully managed. Indeed, the hospital is a hostile environment for the oldest bodies (17), minds (18), and for the health of intestinal microbes (19), and it is known that the longer the exposures to these iatrogenic hazards the higher the risk of adverse outcomes. The hospital has different routines from the elderly's home, and in-patients have to adapt to meal hours, appointments for physiotherapy, and to an uncomfortable bed while feeling pain and sharing private space with dubious roommates. Furthermore, it is not uncommon that part of the ordered food is left on the plate (20), often due to the lack of appetite or to the unrequited patient's expectations of hospital meal. Irrespective to the hours of physiotherapy per day, the orthopedic patient remains for most of the time in bed, numb from drugs and with no stimuli. Similarly if not worse than at home, the lack of restful sleep causes mood changes and further reduces the desire to move or socially interact (21). If the old patient was malnourished, sarcopenic, osteoporotic, and with sleep debt even before accessing the surgical room, the hospital stay is likely to aggravate the clinical picture. Indeed, it makes sense to aim for accelerated paths. However, some cases require longer stays (e.g., in-hospital rehabilitation) and therefore it is necessary to take measures. In addition to the abovementioned preventive and pre-habilitation programs as far as it is concerned for elective orthopedic surgery, the feasibility of early supervised nutritional (22), motor (23), and sleep care (24) was already suggested for improving outcomes in hospitalized patients. Certainly, the more extended is the contact with the patient the more information, education, and therefore reassurance can be conveyed. However, many contextual barriers can frustrate the adherence to hospital-based educational programs, such as the not uncommon post-operative delirium that reduces the attention and increases the disorientation of the old patient (25) or the general resistant to change of clinicians. The adaptation to fit local settings (e.g., areas for motor activity), the redevelopment of hospital systems (e.g., meal ordering system, beds), and the presence of a full-time specialized staff are just some of the facilitating factors for efficacious integrations of innovative protocols nevertheless (26).

## “Home Alone” (Chris Columbus, 1990)

There is little use in feeding healthy food without teaching to cook, in exercising without giving autonomy to movements, and in providing rest without guaranteeing the same conditions at home. The home of the elderly can be considered, similarly but in some ways with less hazards than hospital, a hostile environment. The surgical procedure may have certainly improved the joint pain but the home-based routine is still scattered with risks of malnutrition, falls, insomnia, and solitude as soon as the patient is discharged. Frequent add-on broad-spectrum conditions like the geriatric anorexia (27), inflammaging (28), and immunosenescence (29) may be present, being frequently associated with a basal malnutrition and immunoincompentence (30). Readmissions figures from surgical/medical causes are not scarce (31) and only a small percentage may be potentially preventable (32). Intrinsic factors are numerous nevertheless, counting a history of fall, polypharmacotherapy, and age-related physical and mental decline (33). This unescapable phenomenon emphasizes the need for an effective integration between preventive, treatment, and rehabilitation paths, as it appears that one cannot discern from the other. Home-based nutritional (34), motor (35), and sleep programs (36) were already suggested to be worthy of being considered as effective other than safe. Certainly, attentive medical information and monitoring for increasing adherence is a critical success factor as the educational nature of HEPAS is the genuine backbone of the whole approach. Its principles must be taught and perceived not as temporary interventions but as definitive long-term changes. Life planning after Orthopedics is unique not only to those who have a whole life ahead (37) but also to those who have been on the other side of the path (38).

## “Unbroken” (Angelina Jolie, 2014)

**Figure 1:**
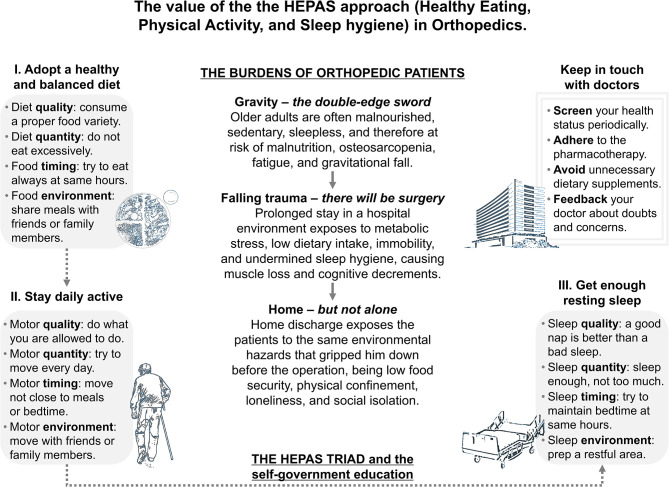
The value of the HEPAS approach (Healthy Eating, Physical Activity, and Sleep hygiene) in Orthopedics. Each phase of musculoskeletal disease progression and care path is known to be stumbling upon deficits of nutritional, motor, and night-rest nature, eventually leading to falling traumas or orthopedic surgery. The HEPAS approach brings together indications on healthy eating, physical activity, and sleep hygiene. This triad, completed by an appropriate self-government education, may be the ideal candidate program to support individual health both in the home environment and institutionalized settings.

The elderly are the most fragile segment of the population and are the first to pay when the health system is severely tested (10, 39). Indeed, older adults are the individuals who suffer the most from nutritional, motor, and sleep deficits nevertheless, easily stumbling upon the double-edged blade of gravity, emergency procedures, hospital hazards, inauspicious recovery, and risk of long-term disability (10). State-of-the-art orthopedic procedures fix what was previously broken. However, it is not only the joint that needs to be reconstituted but also the perception of society toward what is old and slow. Nourishment, movement, and rest are primary needs for an old person's quality of life and they are not least associated with the number of years nevertheless. The HEPAS could succeed in taking care of older people because its dimensions precisely fill the recurrent educational gaps that accelerate the para-physiological decline. Integrated with a self-government education that balances the doctor-patient relationship, perhaps the HEPAS itself can be the valuable means to finally slow down (see Figure 1). It is also important to consider that older adults progressively lose the ability to clearly think, learn, and remember. A cognitive enhancement program *via* environmental enrichment (e.g., creation of reliable social networks and digital systems), in contrast to the electric (e.g., neural prostheses or non-invasive neuromodulation) and drug enhancement, may be useful in complementing the behavioral triad (40). In parallel, the health system could breathe a sigh of relief as it is constantly focused on treating the consequences while missing the causes, sooner or later being unable to qualitatively assist the growing number of incapacitated seniors (41, 42). Taking care of the deficits that affect muscles, bones, and the general physical and mental ability, the HEPAS could have value in musculoskeletal health promotion, prevention of osteosarcopenia and falling traumas, optimization before elective orthopedic surgery, post-operative rehabilitation, and home-base life sustenance. In the future, assuming that HEPAS can be applied for different musculoskeletal-related conditions, it will be necessary to detail the adaptation of each dimension to patient types (e.g., at risk of vs. with disease) and phase (e.g., prevention vs. rehabilitation). Confidently, telemedicine approaches will favor the inclusion of valid remote programs (43), managing home-based individuals through interactive platforms capable of reviving the “Eternal Sunshine of the Spotless Mind” (by Michel Gondry, 2004).

## Author Contributions

MB formulated the opinion, wrote the first draft, contributed to sections, and approved the submitted version of the manuscript.

## Conflict of Interest

The author declares that the research was conducted in the absence of any commercial or financial relationships that could be construed as a potential conflict of interest.
